# Delayed Lymphatic Reconstruction for Breast Cancer-Related Lymphedema

**DOI:** 10.3390/cancers18182934

**Published:** 2026-09-10

**Authors:** Judith Monzy, Jocelyn Lu, Ara A. Salibian, Philip S. Brazio, Ketan M. Patel

**Affiliations:** 1 Division of Plastic and Reconstructive Surgery, Department of Surgery, Cedars-Sinai Medical Center, Los Angeles, CA 90048, USA; judith.monzy@students.jefferson.edu (J.M.); jocelyn.lu@csmc.edu (J.L.); philip.brazio@csmc.edu (P.S.B.); 2 Division of Plastic and Reconstructive Surgery, Department of Surgery, UC Davis Medical Center, Sacramento, CA 95817, USA; ara.salibian@ucdavis.edu

**Keywords:** breast cancer-related lymphedema, lymphovenous bypass, vascularized lymph node transfer, immediate lymphatic reconstruction, liposuction

## Abstract

Breast cancer-related lymphedema (BCRL) is a chronic disease that stems from damage to the lymphatic system due to breast cancer treatment leading to interstitial fluid buildup in the affected extremity. Patients with axillary lymph node dissection in combination with radiation therapy are at the highest risk of developing lymphedema. The mainstay non-surgical treatment involves participation in complete decongestive therapy with certified lymphedema therapists, daily compression garment usage, at-home pump treatments, and diligent skin care. Surgical treatments options can help alleviate symptoms associated with the disease by improving lymphatic drainage and removing fibrofatty tissue. Immediate lymphatic reconstruction (ILR) involves reconstructing cut lymphatics prophylactically at the time of axillary dissection with lymphovenous bypass (LVB) to decrease the risk of developing lymphedema. Delayed reconstruction addresses clinically diagnosed lymphedema and is divided into physiologic and debulking surgical techniques. Physiologic surgeries include lymphovenous bypass and vascularized lymph node transplantation (VLNT) aimed to treat fluid buildup. Debulking surgeries include lymphatic sparing liposuction or direct excision of fibrofatty tissues in the affected extremity to treat excess fibrofatty tissue secondary to lymphedema. This review discusses the pathophysiology of BCRL, diagnosis and staging of the disease, as well as provides an algorithmic overview on delayed lymphatic reconstruction options for BCRL.

## Simple Summary

Breast cancer-related lymphedema (BCRL) is a chronic condition that can develop after breast cancer treatment and which significantly affects patients’ quality of life. Early recognition and appropriate treatment are important to prevent disease progression and improve long-term outcomes. The aim of this review is to summarize the current understanding of the development, diagnosis and staging of BCRL, as well as the available surgical treatment options. We discuss both preventive approaches, including immediate lymphatic reconstruction, and delayed reconstructive procedures tailored to different stages of the disease. By providing an algorithmic overview of current management strategies, we aim to improve understanding of surgical decision-making and treatment for patients with BCRL.

## 1. Introduction

Breast cancer is the most common cancer among women in the United States with an annual incidence of 30% [1]. Although it remains a leading cause of cancer-related death, breakthroughs in screening and treatment strategies have caused a decline in the mortality rate to 2.3%, according to the National Breast Cancer Foundation [2,3]. As more women outlive their cancer diagnosis, ongoing evidence-based improvements to diagnostic and therapeutic strategies have become crucial to reducing morbidity and improving quality of life among survivors [3].

Surgical management of the axilla has shifted toward de-escalation, guided by landmark trials (Z0011, AMAROS, IBCSG 23-01) [3], favoring sentinel lymph node biopsy (SLNB) over axillary lymph node dissection (ALND) for early-stage breast cancer [4,5,6]. ALND, which is currently indicated for patients with high nodal disease burden, is associated with long-term complications, including unilateral arm pain, numbness, and breast cancer-related lymphedema (BCRL) [7]. Radiation therapy and obesity further increase the risk of BCRL [3]. BCRL is an incurable disease, and although not fatal, can result in lifelong physical and financial disabilities for patients, as well as significant strain on the healthcare system [8]. In this narrative review, we detail the current understanding of the pathophysiology, diagnosis, staging and management of breast cancer-related lymphedema.

### Pathophysiology and Clinical Presentation of BCRL

The lymphatic system is responsible for maintaining tissue fluid and lipid homeostasis, trafficking immune cells and filtering cellular debris [9]. Interstitial fluid enters lymphatic capillaries, travels through precollectors and collecting lymphatics, is filtered at lymph nodes, and re-enters the systemic circulation at the subclavian vein [10].

Disruptions to the normal flow of lymph, including poor capillary filtration, mechanical obstruction of the lymphatic system, and dysfunction of lymphatic propulsion, drive the development of lymphedema [9]. During ALND, it is recommended that approximately 10–20 nodes be dissected, focusing on the level 1 and 2 nodes which are designated according to their location relative to the pectoralis minor muscle [11]. Dissection of level 3 nodes—reserved for cancer that has grossly spread—is associated with an increased risk of BCRL [11]. Although it is known that lymphatic injury contributes to the development of BCRL, not all patients develop lymphedema after such axillary procedures [12]. The incidence of BCRL is around 5% [13], 14% [14] and 33% [14] after SLNB, ALND alone and ALND with radiation therapy, respectively.

In BCRL, obstructed lymphatic flow leads to an upregulation of growth factors that contribute to lymphatic hyperplasia, resulting in the inefficient drainage of lymph and accumulation of protein-rich fluid [15]. Lymphatic fluid stasis facilitates the aberrant activation of naïve CD4+ T-cells, ultimately promoting fibrosis and abnormal lymphangiogenesis [15]. As BCRL progresses, collagen deposition and fibrosis hinder lymphatic vessel contractility and collateral lymphangiogenesis, impairing the flow of fluid from the superficial to the deep lymphatics [15]. Radiation therapy and certain classes of chemotherapeutic agents, such as taxanes, exacerbate the initial insult from ALND, increasing fibrosis formation [16].

Breast cancer-related lymphedema demonstrates significant variability in timing of onset, clinical presentation, and severity among patients. BCRL may present months to years after ALND, with most patients presenting within 2 years postoperatively [8]. While some patients experience a transient postoperative edema, others experience a chronic and progressively worsening lymphedema [8]. The severity of the BCRL varies, ranging from an indolent disease managed with conservative treatments to a disabling, refractory swelling. For patients with chronic lymphedema, the sequelae of BCRL include tingling, redness, tightness, weakness and heaviness in the affected arm [8]. A 2021 prospective study investigating the psychosocial and Health-Related Quality-of-Life (HRQoL) outcomes of BCRL found that the presence of symptoms, regardless of lymphedema status, was inversely associated with HRQoL scores, overall mental well-being, and perceived physical health [17]. The financial burden of BCRL management was also significant: when accounting for productivity losses, those with BCRL incurred 19% higher out-of-pocket costs per year compared to breast cancer survivors without BCRL [18]. For patients with lymphedema, 50% of their total medical expenses were for lymphedema-related treatments [18].

## 2. Preoperative Workup and Diagnosis

### 2.1. History and Patient Assessment

A thorough history is crucial in guiding treatment options. Patients presenting with upper extremity swelling concerning for BCRL require a comprehensive review of their breast cancer treatment history, including prior axillary surgery, radiation therapy, chemotherapy and prior reconstruction [19,20,21]. Risk factors for BCRL include genetic predisposition or a family history of lymphedema, elevated body mass index (BMI > 30 kg/m^2^), cellulitis, and comorbid venous and/or metabolic conditions, including hypothyroidism, cardiac disease, renal disease or deep vein thrombosis [22,23]. The clinical history includes assessment of the timing, onset and location of symptoms, including swelling, heaviness, fatigue, fullness, tightness, pain, numbness, tingling and impaired range of motion [24]. A history of current or prior episodes of cellulitis may precipitate arm swelling [25]. Characterizing swelling as pitting vs. nonpitting, alleviated by elevation and changing throughout the day, helps stage the disease according to the International Society of Lymphology (ISL) classification system which guides treatment [22,26].

Direct examination of the affected extremity includes findings related to limb volume differences between the affected limb vs. the unaffected limb, trophic skin changes, signs of infection and the presence of pitting edema [27]. Swelling of the hand and digits can oftentimes denote advanced lymphedema that is more difficult to treat surgically. Stemmer’s test, which is performed by attempting to pinch the skin at the digit to assess for compliance of skin and extent of fibrosis, is helpful in characterizing the composition of the excess volume as fluid vs. fibrofatty [22,28].

#### Oncologic Considerations

Oncologic treatment deserves an important consideration in BCRL. Systemic therapies may contribute to or exacerbate limb swelling. Taxanes have been consistently identified as independently increasing the risk of arm lymphedema [29]. Taxanes may cause transient extremity swelling, which may complicate the diagnosis of BCRL [30]. Tokumoto et al. suggest waiting at least 6 months after completion of taxane-based chemotherapy before considering lymphatic surgery to avoid operating on reversible edema [31]. Other systemic therapies that might cause more generalized peripheral edema include PI3K, mTOR and CDK4/6 inhibitors. Daniell et al. reported new or worsening edema in 6.8%, 8.8% and 9.2% of patients after taking PI3K, mTOR and CDK4/6 inhibitors, respectively [32]. Surgeons should consider these treatment-related effects when evaluating extremity swelling and determining the timing of lymphatic surgery. Endocrine therapy, while not implicated in causing swelling, increases the risk of arthralgias and musculoskeletal symptoms, which may affect postoperative rehabilitation and adherence to compression therapy [33].

Before proceeding with lymphatic surgery, clinicians should evaluate new or worsening extremity swelling for cancer recurrence and other causes, including deep vein thrombosis [34]. In our practice, surgical treatments are generally reserved for patients without active recurrence or metastatic disease. For patients with metastatic or recurrent disease, the decision to pursue lymphatic surgery should be a shared decision that considers quality of life [35]. 

### 2.2. Diagnosis and Preoperative Planning

**Figure 1:**
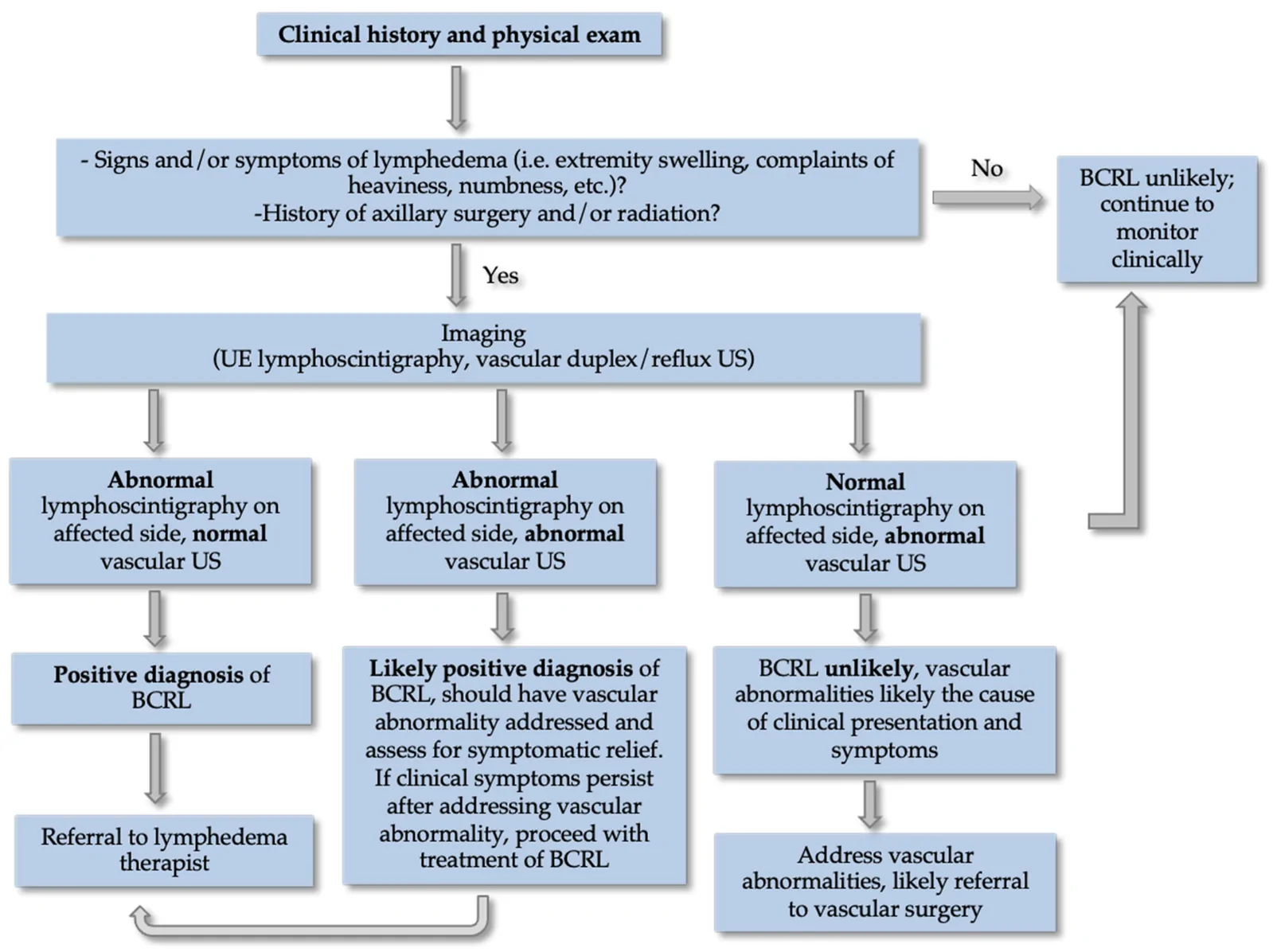
Algorithm for the workup and diagnosis of breast cancer-related lymphedema. Patients presenting with signs or symptoms concerning for BCRL undergo clinical assessment followed by lymphatic and vascular imaging when indicated. Lymphoscintigraphy and vascular ultrasound findings help distinguish BCRL from vascular etiologies of upper-extremity swelling and identify patients appropriate for referral to lymphedema therapy. BCRL, breast cancer-related lymphedema; UE, upper extremity; and US, ultrasound.

Diagnosis of lymphedema relies on a combination of clinical assessment, limb measurement thresholds and, when indicated, imaging. The differential diagnosis of upper extremity swelling must be worked up before initiating treatment for lymphedema. Deep vein thrombosis (DVT), recurrence of malignancy, external compression or stricture of the axillary vein, infection, congestive heart failure, and renal or hepatic failure are among the differential diagnoses of upper extremity swelling that must be evaluated and ruled out before diagnosis of lymphedema can be made [22,28]. A proposed algorithm for the diagnostic workup of patients presenting with signs or symptoms concerning for BCRL, including assessment for concomitant vascular pathology, is summarized in Figure 1.

#### 2.2.1. Limb Volume Measurements

Obtaining limb measurements is critical for monitoring disease/symptom progression after treatment and determining when volume stabilization has been reached in order to initiate long-term maintenance with compression garments. The most commonly used limb measurement diagnostic thresholds are ≥10% limb volume change, ≥2 cm increase in limb circumference and/or an L-Dex > 10 compared to baseline and/or the contralateral limb [34,36]. There are several methods for obtaining limb volumes:

Water displacement is the gold standard for obtaining limb volumes. It involves submerging a limb in water and measuring the displaced volume [28]. The volume displaced corresponds to the volume of the limb. Several limitations exist for the use of this technique in the clinical setting, based on the time-consuming and cumbersome nature of submerging an extremity [28].

Circumferential measurement, an alternative to water displacement, involves using a tape measure to measure 4, 6, or 10 cm increments along a limb [37]. The truncated cone formula is then applied to obtain limb volumes [37].

Perometry allows for a contactless method of measuring either whole or segmental limb volumes by placing the affected limb in an apparatus with infrared light beams [37]. Cross-sections of the shadow created by the limb are used to derive its volume [37].

Bioimpedance spectroscopy is another commonly used method to assess fluid buildup in a limb in which impedance of the extracellular fluid to an electric current is compared between the affected and unaffected limbs [37]. It is best suited for tracking limb fluid changes and detecting subclinical lymphedema.

#### 2.2.2. Imaging Modalities

##### Lymphoscintigraphy

Lymphoscintigraphy is the gold standard imaging modality used primarily for the diagnosis of lymphedema. A radioactive tracer, technetium-99 m, is injected into the dermis of digits in the affected limb. The tracer follows the flow of lymph and provides qualitative findings such as proximal nodal uptake, visualization of lymphatic ducts, and presence of dermal backflow [38]. It has a sensitivity ranging from 92 to 96% and a specificity of 100% [39]. Lymphoscintigraphy has low resolution and results do not always correlate with severity, making it an appropriate diagnostic tool, but less helpful for treatment planning.

##### Indocyanine Green (ICG) Lymphography

**Figure 2:**
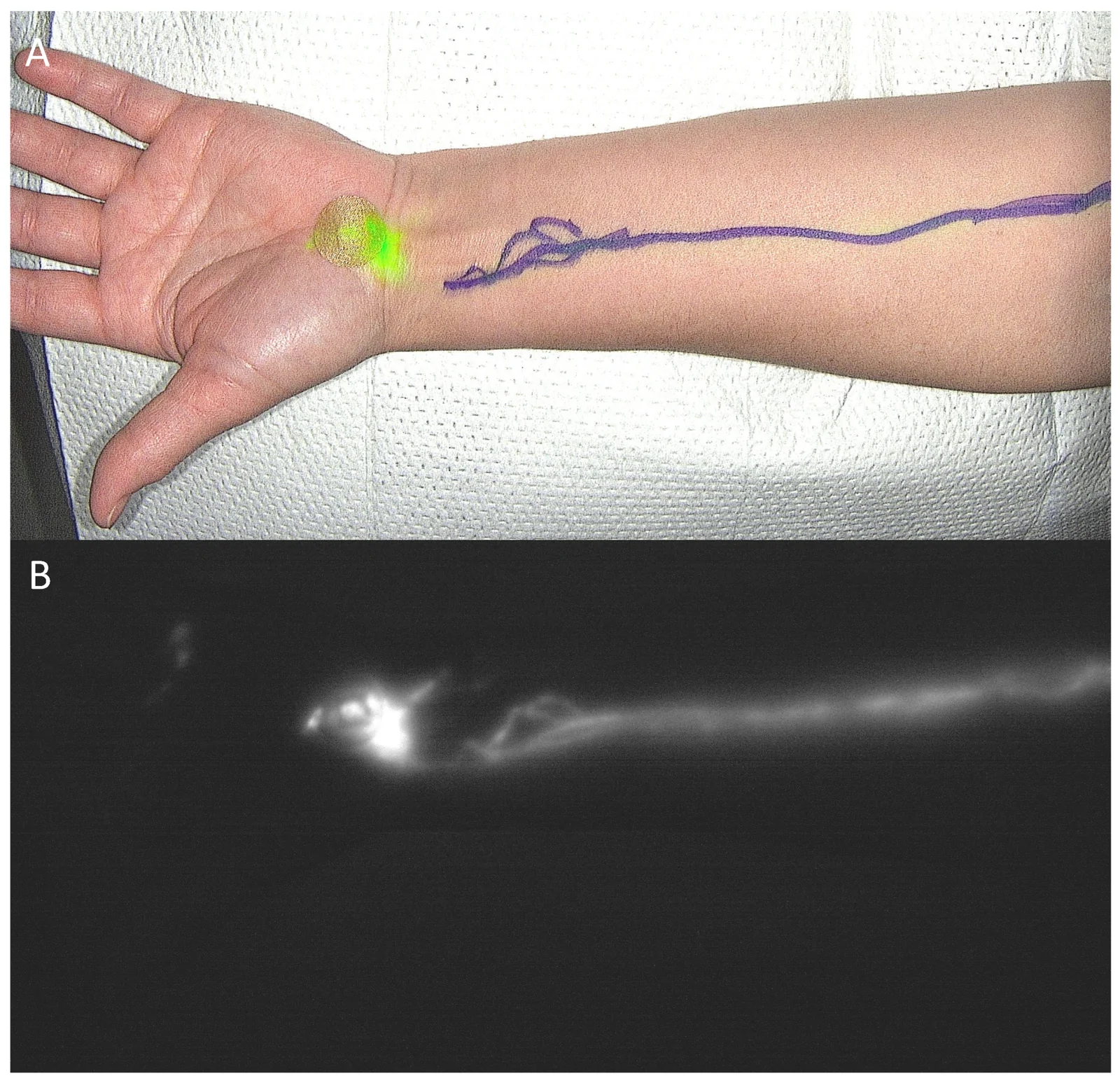
Patient 1 preoperative indocyanine green (ICG) lymphography used for lymphovenous bypass planning. (**A**) ICG (green dye) was injected into the volar wrist with a band aid overtop. A purple marking pen was used to trace out the lymphatic channel traveling proximally towards the axilla. (**B**) Near-infrared fluorescence view of the functional lymphatic channel traveling in a linear pattern. Clinical images are original photographs from the senior authors’ practices.

**Figure 3:**
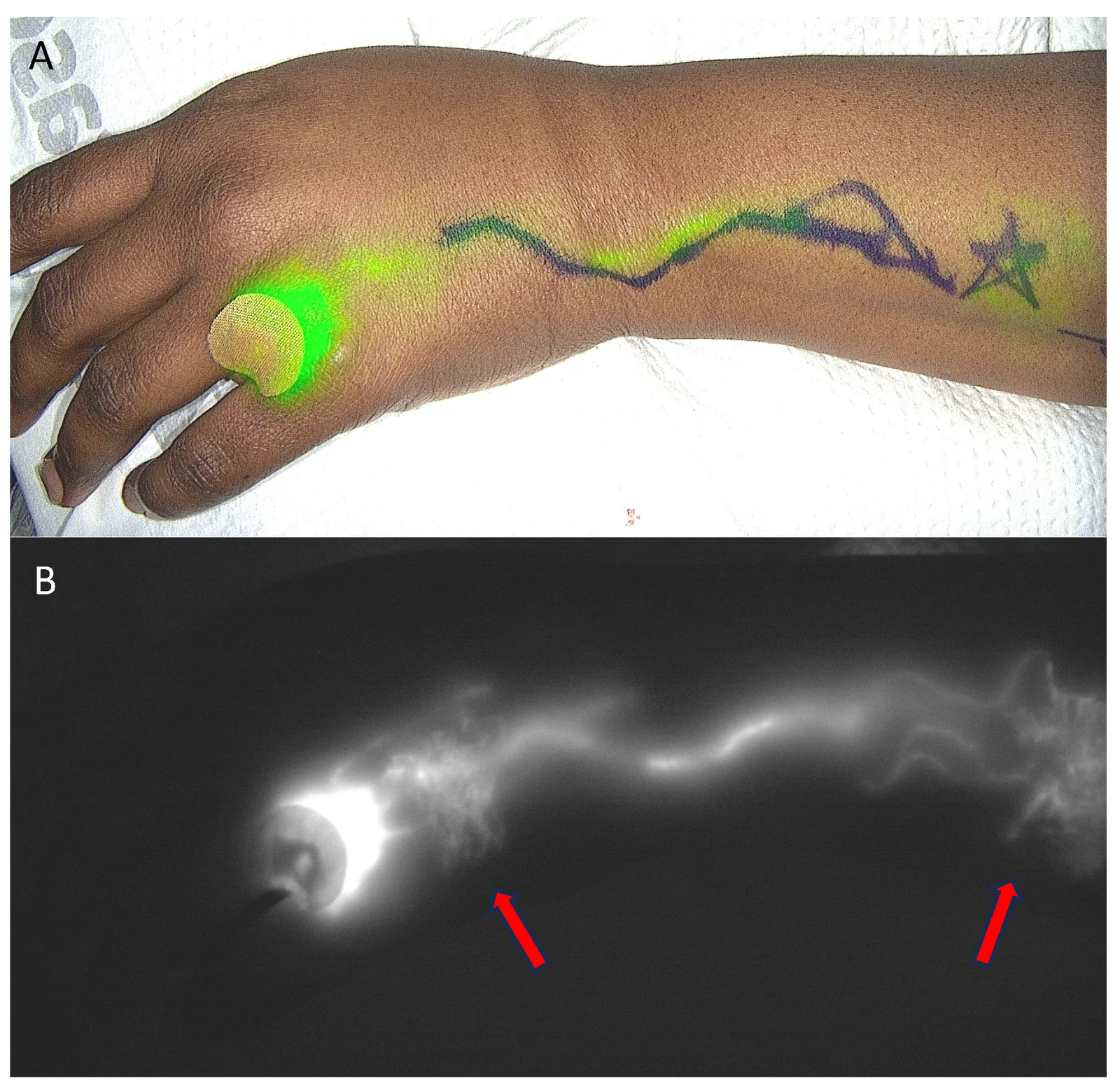
Patient 2 preoperative indocyanine green (ICG) lymphography of the affected upper extremity. (**A**) ICG (green dye) injection was placed in the fourth web space with a band aid covering the injection site. Purple marking was used to trace the lymphatic flow in real time on the forearm. A star was placed at the site of early dermal backflow. (**B**) Near-infrared fluorescence view demonstrating the lymphatic vessels with some linear flow and areas of early dermal backflow proximal and distal to the injection site (red arrows). Clinical images are original photographs from the senior authors’ practices.

**Figure 4:**
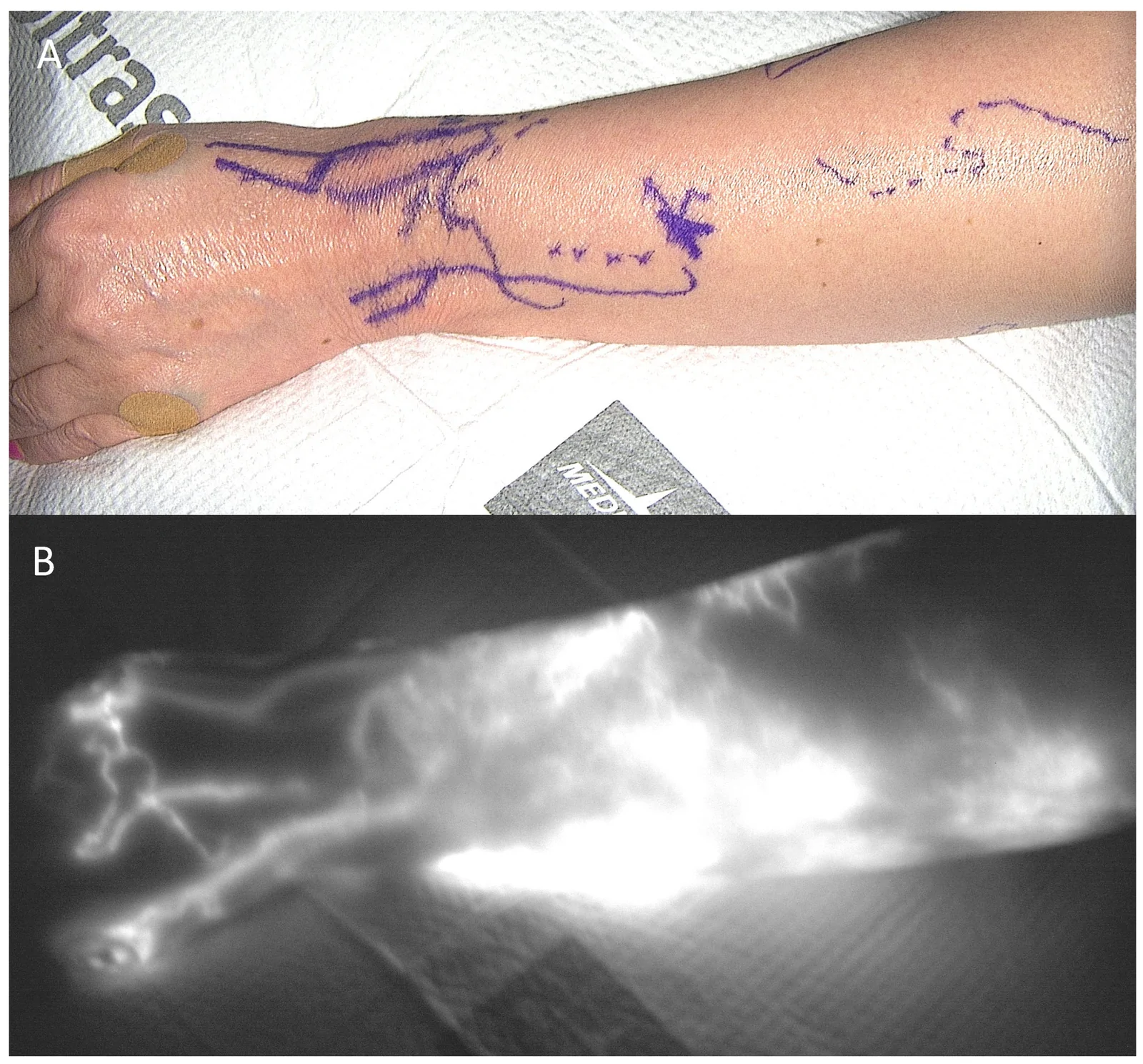
Patient 3 preoperative indocyanine green (ICG) lymphography of the affected upper extremity. (**A**) ICG was injected in the first and fourth web spaces with band aids applied overtop. A star is placed at the site of a linear channel ending in dermal backflow. (**B**) Near-infrared fluorescence view demonstrating diffuse (stardust/splash patterns) throughout the dorsum of the forearm. Clinical images are original photographs from the senior authors’ practices.

Indocyanine green (ICG) lymphography is a widely used imaging modality that allows surgeons to observe lymphatics in real time to identify areas of dysfunction. It is useful pre-, intra-, and postoperatively for diagnosis, guiding surgical planning (especially during LVB procedures), and postoperative management [38]. A water-soluble fluorescent dye is injected intradermally and a handheld near-infrared camera may be used to visualize lymphatic channels as well as areas of backflow secondary to lymphatic dysfunction [38]. It has a sensitivity and specificity of 97% and 92%, respectively [40]. Like lymphoscintigraphy, intact lymphatics are seen as linear patterns (Figure 2), while leaking and dysfunctional lymphatics present with splash, stardust, or dermal backflow patterns (Figure 3 and Figure 4) [41]. ICG lymphography is limited to visualization of the superficial lymphatic system. Individuals with iodine allergies are at risk of allergic reaction, including anaphylaxis, as ICG contains sodium iodide.

##### Magnetic Resonance Imaging Lymphography (MRL)

Magnetic resonance imaging lymphography (MRL) is a noninvasive imaging modality that helps visualize the superficial lymphatics for patients with lymphedema [42]. MRL, compared to lymphoscintigraphy, allows for 3D visualization of the lymphatics without using a radioactive tracer. MRL can also image late-stage lymphedema, showing adipose deposition and fibrosis [42]. Dayan et al. demonstrated that MRL is an effective technique to assess fluid vs. fat components in lymphedema extremities [43]. These findings showed that patterns of fat accumulation correlated with the ISL stage, with more advanced stages associated with higher fat grades [43]. MRL has a 92–100% sensitivity and 100% specificity in revealing lymphatic drainage patterns and abnormal lymphatic vessels [44].

##### Ultra-High-Frequency Ultrasound (UHFUS)

Ultra-high-frequency ultrasound (UHFUS) utilizes higher frequency ultrasound probes to visualize the superficial lymphatics and venules of affected limbs in real-time. It is especially helpful in preoperative and intraoperative planning of LVB [45] as it allows for direct real-time visualization of lymphatic channels as well as assessment of lymphatic channel size and degree of vessel sclerosis. Hayashi demonstrated that UHFUS can capture functional lymphatics and those as small as 0.3 mm in diameter [45]. Bianchi found substantial agreement between the UHFUS and histologic findings of lymphatic vessels when evaluating lymphatic vessel degeneration status in patients with lymphedema [46]. This agreement was observed when comparing the inner diameter, outer diameter, and wall thickness measurements of lymphatic vessels using the two methods [46]. Hayashi’s study revealed a sensitivity of 94.9% and a specificity of 98.3% of UHFUS for detecting lymphatic vessels [45]. Ultimately, UHFUS, along with ICG and SPY-PHI, allows surgeons to identify venules near functional lymphatics to aid in preoperative planning and identify target sites for bypass on the extremity.

### 2.3. Staging

Staging lymphedema may be separated into clinical and image-based staging systems. The ISL staging system is most widely used for classifying lymphedema. Stage 0 represents subclinical changes identifiable on imaging. Stage I involves reversible swelling with pitting edema. Stage II presents with persistent pitting unrelieved by elevation, [47] and stage III is characterized by fibrofatty and trophic skin changes indicating severe lymphedema [47]. Another clinically relevant staging system is one developed by Campisi that incorporates limb appearance and symptomatology based on the patient’s clinical picture and lymphoscintigraphy results [48]. Stage 1A represents subclinical changes detectable on lymphoscintigraphy, and stage 1B involves mild edema reversible by limb elevation [48]. Stage 2 is persistent edema occasionally reversible by limb elevation [48]. Stage 3 is persistent edema with recurrent infections called erysipeloid lymphangitis [48], stage 4 is the presence of a column-shaped limb due to marked fibrosis [48], and stage 5 is elephantiasis with severe limb deformity [48].

Imaging-based classification systems are helpful in visualizing the lymphatics for preoperative and/or intraoperative surgical planning. ICG lymphography has helped guide lymphedema staging and classification systems based on patterns of dermal backflow. The MD Anderson lymphedema classification system is divided into six stages based on the extent of dermal backflow, vessel patency, and contractile function seen on ICG and follows the progression of lymphedema [40]. Stage 0 demonstrates normal lymphatic function with patent lymphatic vessels, no dermal backflow, and normal contractility. Stage 1 demonstrates several patent lymphatics with minimal dermal backflow. Stage 2 shows a moderate number of patent lymphatics and moderate dermal backflow. Stage 3 reveals few patent lymphatics and severe dermal backflow, stage 4 shows dermal backflow at the hand, and stage 5 represents severe lymphedema with no vessel patency, no dye movement, and absence of contractility [40].

## 3. Treatment of Lymphedema

The decision-making process for lymphedema treatment is guided by several factors including symptom severity, presence of comorbidities, patient-related factors and most importantly, disease stage based on fat-fluid ratios as well as the degree of lymphosclerosis progression. The goals of lymphedema treatment are to reduce symptoms, stabilize limb volume reduction and decrease the frequency of infections. BCRL treatments may be divided into conservative (non-surgical) and surgical approaches. The foundation of non-surgical treatments involves daily compression garment use, physical therapy, risk-reduction and skin care [28]. As a chronic disease without a known cure, non-surgical treatments are the best defense for slowing the progression of BCRL. Surgical treatments may be offered to reduce patient burden and further slow disease progression.

### 3.1. Non-Surgical Approaches

#### 3.1.1. Complete Decongestive Therapy (CDT)

Complete decongestive therapy remains the mainstay for the initial management of BCRL [47]. CDT is a two-stage treatment program performed by a certified lymphedema therapist. The first stage is the reduction phase, during which manual lymphatic drainage (MLD), skin care, exercise, bandaging and patient education are employed to reduce limb volume and manage symptoms. MLD is a massage technique that facilitates lymph flow, skin care assesses for the onset of cellulitis or infections, exercises allow for effective lymphatic pumping, compression involves short-stretch bandaging and garments custom-fitted to the patient, and patient education emphasizes employing risk-reduction strategies [24,49]. The first stage of CDT may be performed for up to two hours nearly every day over several weeks until optimal limb volume reduction and symptom control are achieved. This is followed by the maintenance phase, which consists of skin care, exercise, and daytime and overnight compression garments worn as much as is tolerable [47,50]. The first-line treatment for ISL stage 0 and stage I lymphedema is patient education, massages, exercise, and/or use of fitted compression garments [51]. CDT is recommended for cases of progressive lymphedema. Dayes et al. reported a significantly greater absolute volume loss in patients who received CDT compared to those who only received compression therapy, although the mean reduction in excess arm volume was not statistically significant [52]. Quality-of-life scores did not differ significantly between the two groups [52]. Kavak and Ünver reported a significant decrease in upper limb volume (393.5 mL, *p* < 0.001), ISL stage (2 to 1, *p* < 0.001), disability-related quality-of-life score (Quick DASH) (40.91, *p* < 0.001) and an increase in health status assessment (40, *p* < 0.001) in patients with BCRL after CDT [53].

#### 3.1.2. Compression Therapy

Compression therapy is an effective long-term strategy to control the progression of lymphedema and works by promoting lymphatic fluid reabsorption and improving tissue quality. By applying mechanical forces, it increases interstitial pressure, enhances lymphatic pumping, and modulates cellular activity within fibrosclerotic tissue [54]. Compression bandages and garments are worn both with conservative therapy and postoperatively to maintain volume reduction. Both are indicated for all stages of BCRL and are often used to maintain the effects of MLD [28]. Compression bandages mimic the physiologic propulsion of the lymphatics by providing a resting pressure during the relaxed state and an opposing working pressure during activity [28]. Compression garments apply longitudinal and transverse pressures when worn on the arm, with the greatest pressure above the wrist [28]. Compression gloves may be worn, along with a full sleeve, to prevent dermal backflow into the hand [28]. The STRIDE algorithm, developed by Bjork and Ehmann, provides a comprehensive and standardized approach to compression garment selection beyond dosage alone [55]. STRIDE considers the interplay of lymphedema characteristics, patient-specific considerations and garment features to individualize therapy.

Compression garments can be categorized into circular-knit, stiffer circular-knit, flat-knit, adjustable wraps, decongestive wraps and night-time garment types [55]. Circular-knit garments are soft, thin and highly elastic, providing similar resting and working pressures [56]. They are best suited for patients with mild edema and are designed for patient comfort, making them less effective in limbs with complex contours [56]. Stiffer circular-knit garments provide better edema containment with moderate to high compression and may be layered to augment compression [56]. Flat-knit garments are constructed from thicker yarns, offering more stiffness and providing more dynamic compression and more uniform pressure distribution [56]. They better accommodate complex contours, resist rolling with custom design and are preferred for moderate to severe lymphedema [56]. Adjustable wraps are stiffer than flat-knit and circular-knit garments and provide more dynamic compression [56]. Compression may be modified by the user, making it more suitable for the acute phase of lymphedema management [56]. Decongestive wraps share similar textile properties with adjustable wraps and are commonly used during the reduction phase of CDT, either in addition to or in place of multilayer short-stretch bandaging [56]. Night-time garments provide less compression while prioritizing patient comfort, and may be selected according to the patient’s swelling characteristics and overall compression needs [56].

Pneumatic compression pumps represent another category of compression therapy where the affected limb is placed in an inflatable garment that applies rhythmic pressure to the limb to decrease lymph retention [24]. Daily use of pneumatic compression pumps facilitates fluid movement from the affected extremity and helps reduce fluid accumulation. Badger reported a 32.6% reduction in limb volume in patients with unilateral lymphedema who had compression therapy and CDT compared to a 19.6% reduction in patients who only applied compression therapy after 24 weeks [57].

Before undergoing any surgical treatment for lymphedema, patients typically partake in CDT with adherence to therapy sessions, home care and compression garment use for at least 3–6 months. This is important to maximize fluid removal from the limb to help optimize surgical success, as well as to ensure patients can receive adequate therapy in a timely fashion in the perioperative phase.

### 3.2. Surgical Approaches

Surgical treatments for lymphedema may be broadly categorized into immediate and delayed techniques, with immediate lymphatic reconstruction serving as a prophylactic approach, while delayed surgical options aim to provide therapeutic improvement in symptoms. Delayed lymphatic surgeries can be further divided into physiologic and reductive treatments [22]. Physiologic procedures, as the name implies, are aimed at improving the physiologic function of the lymphatic system to combat fluid overload, while reductive treatments are aimed at treating symptoms by reducing limb volume due to excess fibroadipose tissue without addressing the underlying cause of excess volume [22].

**Figure 5:**
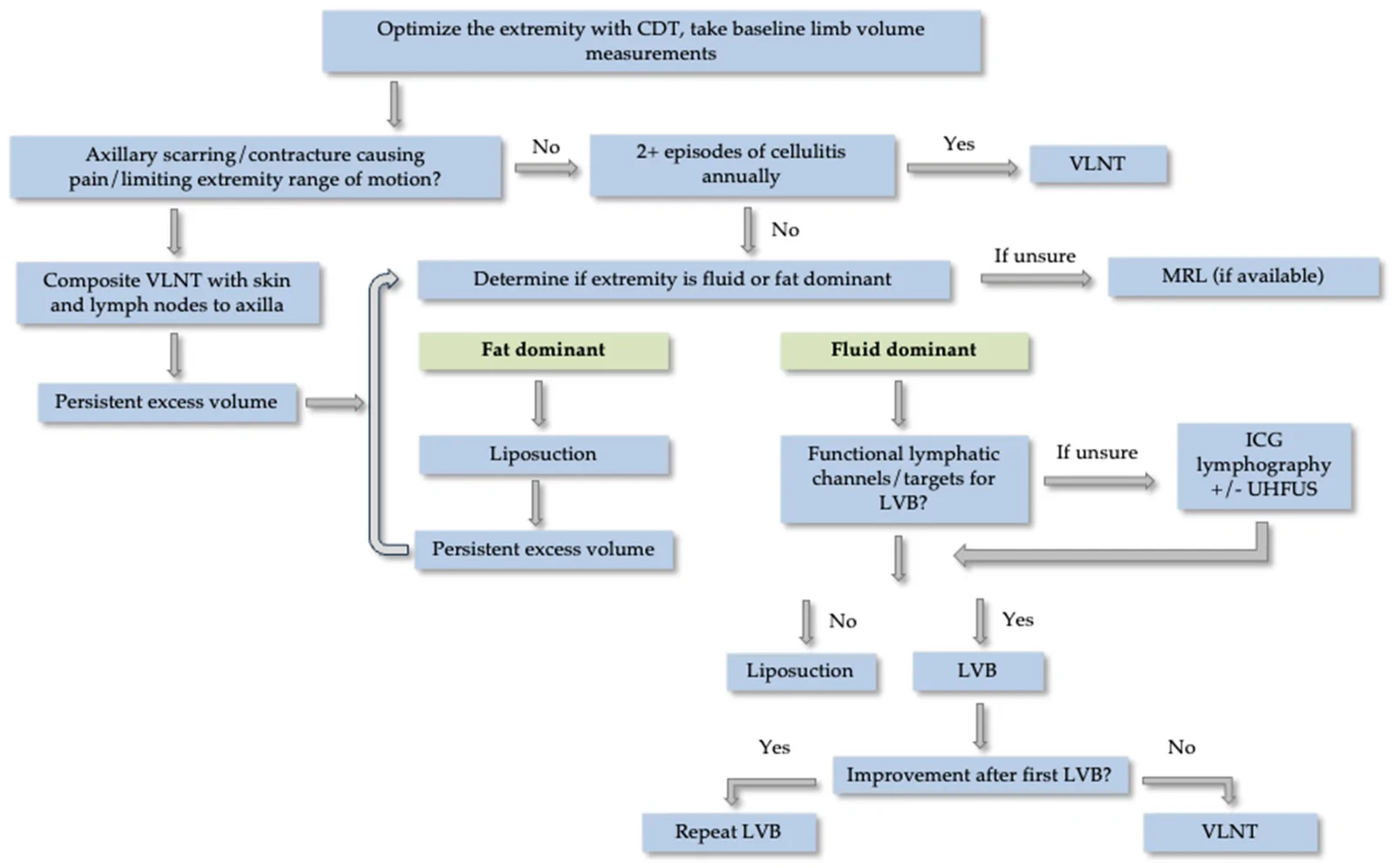
Algorithm for surgical management of breast cancer-related lymphedema. BCRL, breast cancer-related lymphedema; CDT, complete decongestive therapy; ICG, indocyanine green; UHFUS, ultra-high-frequency ultrasound; LVB, lymphovenous bypass; VLNT, vascularized lymph node transfer; and MRL, magnetic resonance lymphography.

Delayed surgical intervention is generally indicated for patients who have trialed and been compliant with conservative therapy for 3 to 6 months and have plateaued with regard to symptom improvement, and those with recurrent episodes of cellulitis. While clinical staging systems are helpful at determining the need for physiologic surgery versus reductive surgery, imaging such as ICG lymphography, UHFUS and MRL can help guide the surgical approach by assessing the composition of excess volume and visualizing lymphatic anatomy, as well as the severity of lymphatic disruption [26]. Physiologic treatments include lymphovenous bypass (LVB) and vascularized lymph node transfer (VLNT) and are typically indicated in patients with a high fluid burden [26]. Reductive procedures include liposuction and direct excision of fibrofatty tissues and/or affected skin., They are best suited for extremities with high adipose-to-fluid ratios in the limb or severely advanced disease in the latter case of excision [26]. It is important to note that these procedures are not curative and require long-term management and compliance with conservative therapies [22]. Following optimization with conservative therapy, selection of a surgical strategy is guided by tissue composition, functional lymphatic anatomy, recurrent cellulitis, response to prior lymphatic procedures, and patient-specific anatomic considerations, as summarized in Figure 5.

#### 3.2.1. Preoperative Optimization

After diagnosing an individual with BCRL, the decision to surgically treat the patient requires a thorough preoperative evaluation. The goals of surgical treatment, whether they be physiologic or excisional procedures, are to mitigate disease progression and limit the morbidity associated with BCRL [58]. A prerequisite to surgical treatment is trialing conservative therapy. As previously mentioned, CDT is the first-line treatment for lymphedema and must be trialed for at least 6 months [58]. Surgical management is pursued if patients demonstrate progression of disease despite CDT or if the disease morbidity is not amenable to conservative treatments, such as patients with recurrent cellulitis and symptoms causing significant psychosocial distress and/or disability [58]. Patients who have active cellulitis or infections must be treated with antibiotics and must be infection-free prior to pursuing surgical treatment [22]. As with any elective surgical procedure, careful patient selection and preoperative medical optimization are important for minimizing perioperative morbidity. Modifiable comorbidities and risk factors should be optimized prior to surgery, and active cellulitis or other infections should be adequately treated before proceeding with elective lymphatic reconstruction. Procedure-specific risks and donor-site morbidity should also be considered when selecting the appropriate reconstructive approach.

#### 3.2.2. Immediate Reconstruction

##### Immediate Lymphatic Reconstruction (ILR)

Immediate lymphatic reconstruction (ILR) is a preventative procedure pioneered by Boccardo et al. that involves performing a lymphovenous anastomosis at the time of ALND [59]. Boccardo demonstrated a 4-year follow-up lymphedema rate of 4% after ILR [59]. Since its adoption, the rate of lymphedema after ILR has ranged from 4.8 to 9.5% compared to 23.6–34% after ALND alone [60,61,62].

#### 3.2.3. Delayed Reconstruction

##### Physiologic Techniques

In general, patients with early-stage lymphedema (stages I–II) and those who have predominantly fluid-excess swelling are ideal candidates for physiologic surgical procedures.

1. Lymphovenous Bypass (LVB)

Lymphovenous bypass (LVB) is performed to circumvent lymphatic obstruction and return lymph back to the venous system. It involves rerouting a functioning lymphatic vessel that terminates in an area of dermal backflow to a nearby vein or venule, as identified by ICG lymphography. Intraoperative considerations include selecting lymphatics with minimal sclerosis and venules with functional valves to prevent reflux [63]. LVB may be better suited for earlier stages of lymphedema compared to later stages as sclerotic vessels are more difficult to anastomose with thicker walls yet smaller intraluminal diameter, and may present with flow issues due to increased interstitial pressures [64]. Successful LVB requires the presence of functioning lymphatic channels, typically detected for ICG, and is therefore traditionally better-suited for early stages of lymphedema.

It is also important to evaluate the axillary vein for stenosis, extrinsic compression, or perivascular scarring that may impair effective lymphatic drainage when performing a lymphovenous bypass (LVB) [65]. To optimize LVB outcomes, lymphatic channels are bypassed into a low-pressure vein to allow the flow of lymph from the lymphatic channel into the vein along a favorable pressure gradient. High pressure within the vein or venous reflux can cause reflux of blood into the lymphatic and impair forward flow of lymphatic fluid. Addressing extrinsic axillary compression or releasing perivascular scar tissue relieves stenosis and unwanted increased pressure on the lymphatics. Studies evaluating perivascular scar release of the axillary vein performed at the time of LVB have shown that this significantly improves lymphedema outcomes by restoring the pressure gradient that normally drives lymphatic outflow into the venous system [65].

In the operating room, small aliquots of reconstituted ICG are injected intradermally into the web spaces of the hand [22]. Isosulfan blue dye can also be injected intradermally in the web spaces and along the extremity to aid in direct visualization of the lymphatic channels intraoperatively [22]. A near-infrared handheld camera is used to visualize the ICG as it is taken up by the superficial lymphatics and travels proximally up the extremity towards the axilla [22]. The lymphatic pathways are marked with a skin marker. If the dye does not spontaneously travel proximally, a lap sponge or towel can be used to help milk the skin to propel the ICG along any potentially patent lymphatic channels. The ultimate goal with lymphatic mapping is to find a linear lymphatic channel that terminates in a patch of dermal backflow, including a proximal obstruction, as this will be a target site for potential lymphovenous bypass.

**Figure 6:**
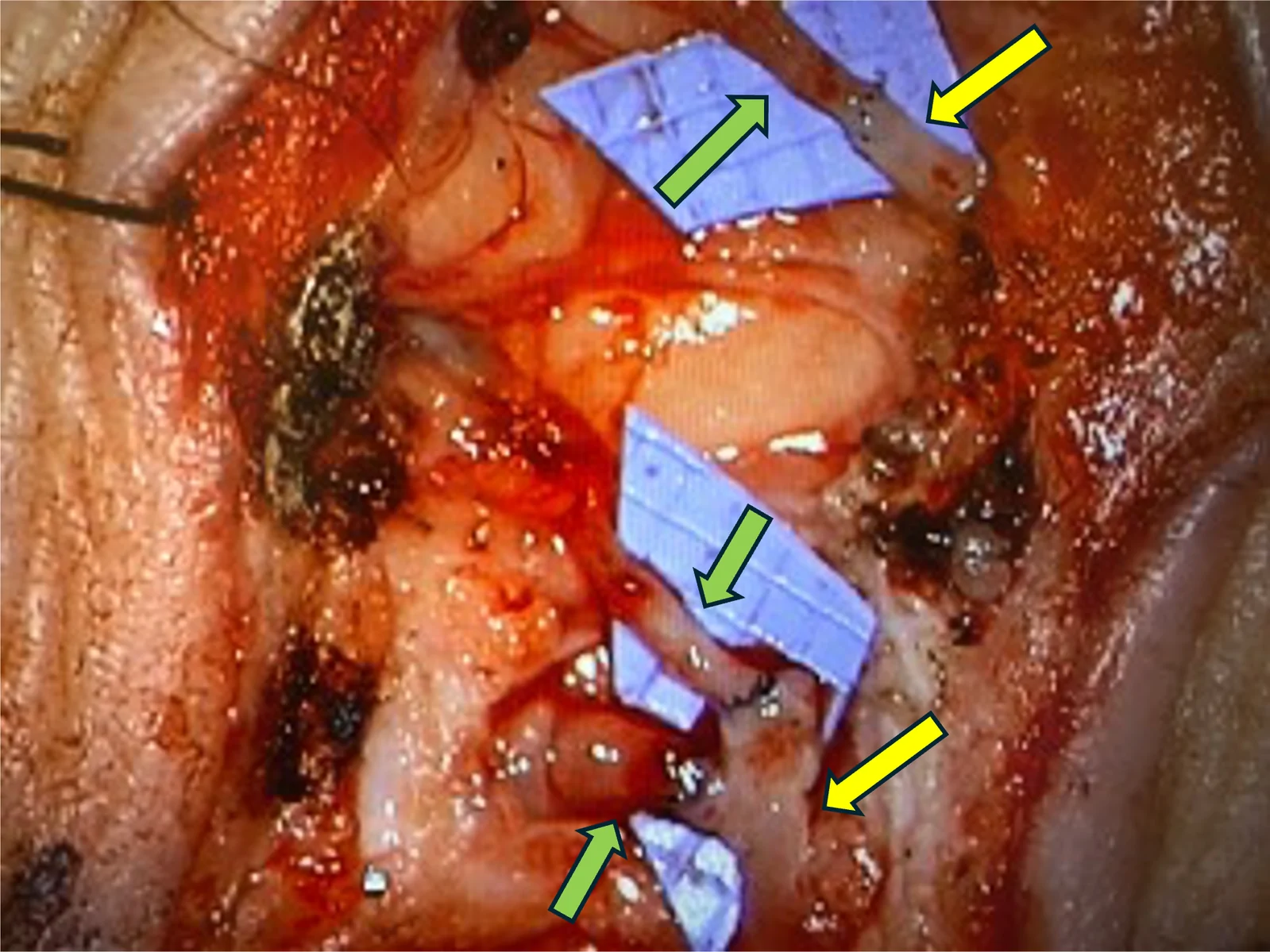
Patient 4 intraoperative photograph of multiple lymphovenous bypasses performed in end-to-end configurations. Lymphatic channels (green arrows) are anastomosed to adjacent recipient veins (yellow arrows). The background grids contain 1 × 1 mm squares for scale. Clinical images are original photographs from the senior authors’ practices.

**Figure 7:**
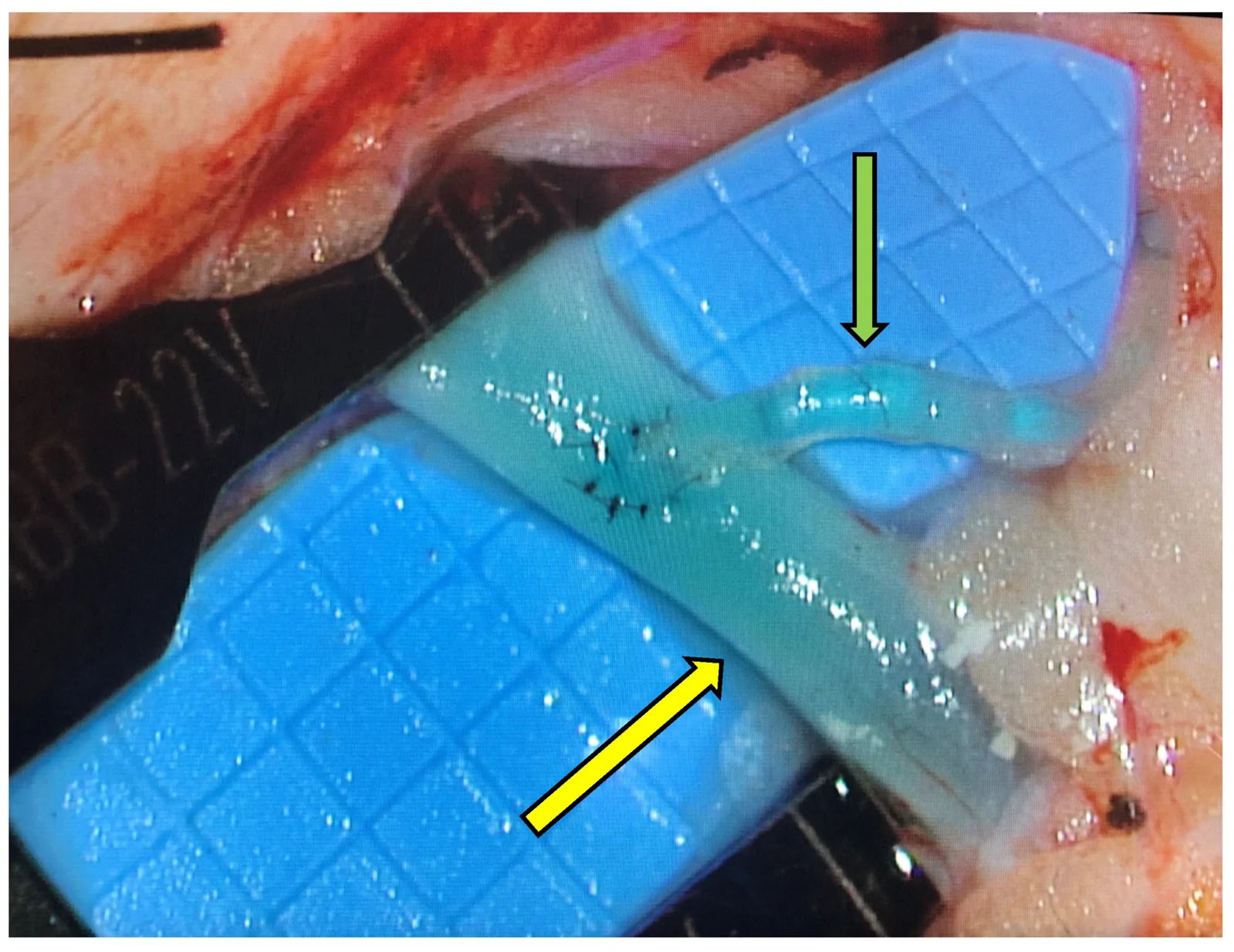
Patient 5 intraoperative photograph of an end-to-side lymphovenous anastomosis configuration. A lymphatic channel (green arrow) is anastomosed to the side of a vein (yellow arrow). The grid background contains 1 × 1 mm squares for scale. Clinical images are original photographs from the senior authors’ practices.

**Figure 8:**
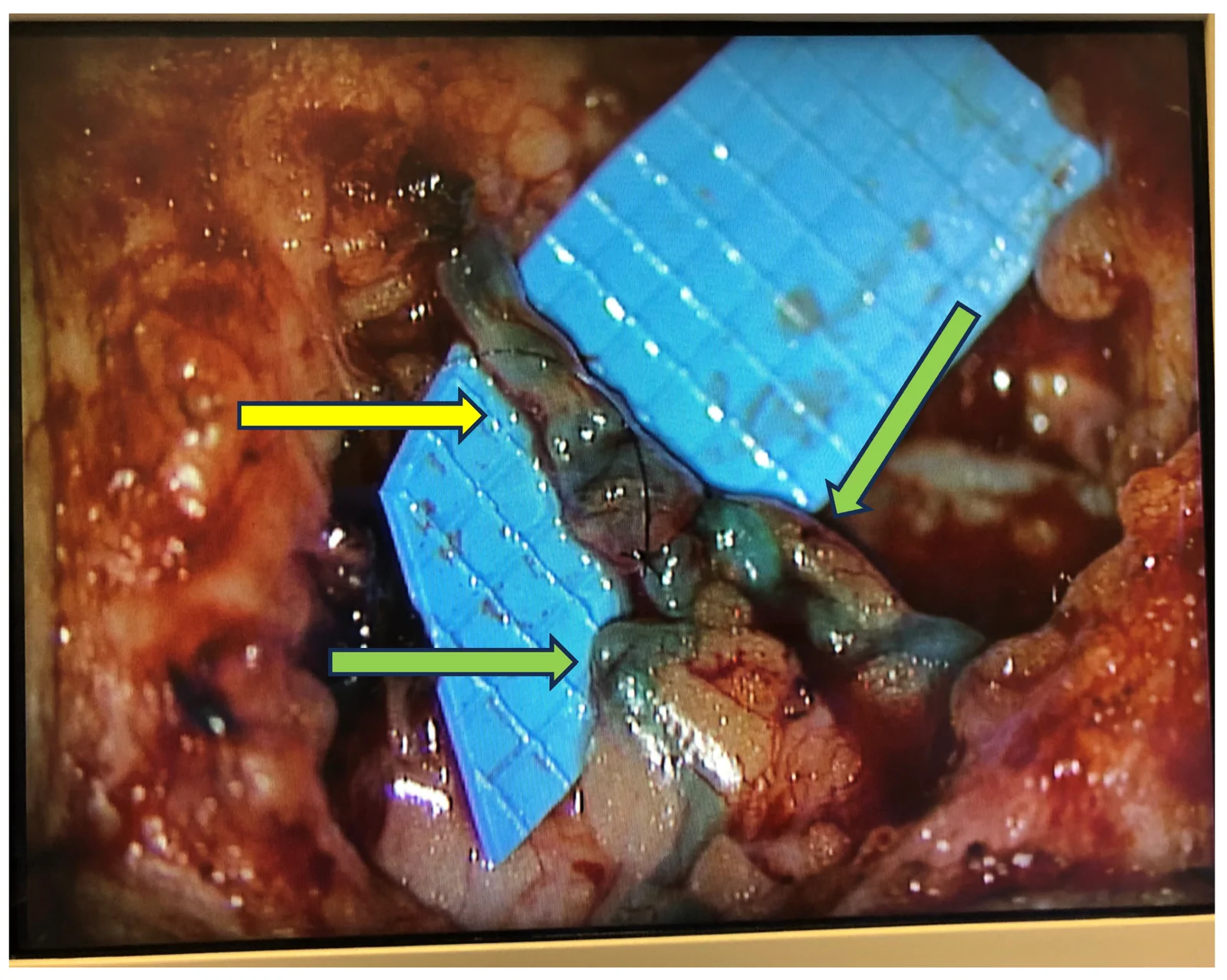
Patient 6 intraoperative photograph of a lymphovenous anastomosis performed using intussusception technique where lymphatic channels (green arrows) are intussuscepted into the distal end of a vein (yellow arrow). The background grid contains 1 × 1 mm squares for scale. Clinical images are original photographs from the senior authors’ practices.

Next, an ultrasound (ideally high-frequency, HFUS), a vein finder, or direct exploration may be used to visualize nearby venules suitable for bypass. A 2 cm transverse incision is made through the dermis overlying the identified target lymphatic, taking care not to incise past the dermis, as the lymphatics and venules may lie very superficially in the upper extremity. The venules are generally found superficial to the superficial fascia and lymphatic channels. If available, UHFUS may also be used to assess lymphatic branching pattern, vessel diameter, and degree of sclerosis. Once a venule and at least one lymphatic is identified, the vein is ligated distally on the extremity, while the lymphatic is cut proximally. Surgical anastomosis may involve one vein to one lymphatic in an end-to-end (Figure 6), end-to-side (Figure 7), side-to-side or double end-to-side techniques [66]. Anastomosis may also involve multiple lymphatics draining into a single vein or may be intussuscepted (Figure 8). Generally, one to four sites may be performed in one surgical event, depending on the number of target channels identified during mapping, and surgeon fatigue. After the skin has been closed and dressings applied, the extremity is wrapped with short-stretch bandaging, and the patient is discharged home with instructions to avoid excess use of the extremity, resume pneumatic pump therapy immediately, and start lymphedema therapy within 3–5 days.

Lymphovenous bypass has been shown to reduce limb volume and improve patient-reported outcomes in patients with BCRL. Koshima, who pioneered supermicrosurgical LVB, reported a decrease in mean excess circumference of 4.1 cm (47.3%) at a mean follow-up of 2.2 years in 12 patients, compared to a reduction of 0.8 cm following conservative therapy alone [67]. Chang et al. reported a mean volume reduction of 35% at 12 months in a cohort of 16 patients, with concurrent subjective symptom improvement [68]. They later reported a 61% reduction in mean limb volume at 1 year in 89 patients with stage I-II lymphedema [69]. Scaglioni et al. described a novel approach of performing an LVB on the deep lymphatics in patients with primary and secondary lymphedema who had suboptimal results after prior superficial LVB [70]. These patients had at least a one-stage improvement in Campisi staging and improvements to both subjective and objective measures of lymphedema [70]. In a cohort of 100 patients, Qiu et al. reported improvement in the upper extremity lymphedema index in 53% of patients, with only 35.5% continuing compression garment use at a mean follow-up of 25 months and a reduction in cellulitis episodes by 0.6 per year [71].

Patient-reported outcomes also improved following LVB. Salgarello et al. reported a 2.43-point improvement in Lymphedema Quality of Life (LYMQoL) after 270 days in 44 patients who underwent LVB for BCRL [72]. Similarly, total Lymph-ICF score, reflecting perceived functional impairment, demonstrated a 30-point decrease at 1 year, indicating symptom improvement [73]. In a randomized control trial, Jonis et al. reported a greater-than-10-point decrease in the domains of physical and mental function after 6 months in those who underwent LVB compared to those treated with CDT alone [74]. Similarly, Qiu et al. reported a 13.3-point reduction in total Lymph-ICF score at a mean follow-up of 25 months [71].

2. Vascularized Lymph Node Transfer (VLNT)

Vascularized lymph node transfer (VLNT) involves free tissue transfer or autotransplantation of vascularized lymph nodes on their vascular pedicle from a donor site to the affected limb. Two primary theories for mechanism of action have been proposed. The regenerative theory states that transferring lymph nodes into a relatively lymph node-depleted region, combined with scar contracture release, promotes lymphangiogenesis [75]. This process is thought to be mediated by lymphangiogenic factors such as VEGF-C [75]. Animal studies have supported this mechanism demonstrating increased VEGF-C expression in nascent lymphatic vessels [76]. This mechanism, however, may be less effective in advanced lymphedema, where lymphatic dysfunction and impaired distal pumping limit the capacity for regeneration [75]. The functional drainage theory suggests that VLNT acts as a low-pressure “sump pump” when placed distal to the lymphedematous region. In this model, gravity facilitates lymphatic flow toward the distal limb, while the “catchman effect” supports the movement of lymph fluid into the flap after which it travels back to the venous system [75,77]. Patients who are better suited for VLNT compared to LVB include those who are at early stages of lymphedema (stages I–II) with fluid-predominance but have very little or no functional linear lymphatics seen on ICG lymphography, and/or those who have recurrent bouts of cellulitis (more than two episodes in 6 months) [22]. Patients with hand and/or foot lymphedema, which represent damaged terminal and/or collateral channels but have fluid-predominant disease, are also better suited for VLNT [26].

Flap choice involves consideration for both donor and recipient sites. Common donor sites used to harvest lymph nodes include the superficial groin, omental, submental, supraclavicular, lateral thoracic and thoracodorsal lymph nodes [22]. These flaps can be harvested as composite flaps that include a skin paddle for flap monitoring and/or wound closure if needed. Ideal donor sites have consistent anatomy, are well hidden, have never been operated on, and have a low chance of developing iatrogenic lymphedema.

In our experience, the superficial groin or omental lymph nodes are first-line for VLNT donor sites. When using superficial groin lymph nodes, reverse lymphatic mapping using dual tracing is imperative to decrease the risk of causing iatrogenic, donor-site lymphedema [22]. Reverse lymphatic mapping during groin VLNT harvest was first described by Dayan, Dayan and Smith as a method to isolate and harvest only the nodes that drain the trunk [78]. Dayan explains that the conventional teaching of using anatomic landmarks, such as the inguinal ligament and groin crease, to identify desired nodes fails to account for the variability of lymphatic pathways among patients [78]. Following Dayan’s described technique, technetium-99 (Tc99) is injected into the pedal web spaces ipsilateral to the inguinal donor site, and a gamma probe is used to identify the lymph nodes draining the lower extremities [78]. These nodes are marked and are avoided. ICG is then injected along the lower abdomen above and along the inguinal ligament to identify nodes draining the abdomen [78]. Care is taken to ensure that the harvested flap contains only lymph nodes that are ICG-positive and Tc99-negative, indicating that they drain the abdominal wall rather than the lower extremity [78]. Ultimately, intraoperative ICG and Tc99 evaluation ensures that the flap has enough perfusion through its vascular pedicle and the nodes that drain the lower extremity are preserved [78].

Recipient site selection along the affected extremity is typically surgeon-dependent but is based on two principles. Orthotopic transfer involves transferring the lymph node flap to the primary site of lymphatic obstruction (in BCRL, the axilla) to re-establish drainage [79]. An advantage of this approach in patients who have undergone extensive axillary dissection and radiation is that VLNT introduces healthy, well-vascularized tissue into the region and allows for simultaneous scar release and soft tissue resurfacing for patients with hollowing, tethering and/or contractures [80]. This has been shown to reduce contracture and pain, while improving cosmetic outcomes after aggressive local treatment [80]. Heterotopic transfer, on the other hand, involves transferring the lymph node flap to the forearm, at a site distal to the site of obstruction to drain distal pooling [79]. While distally transplanted flaps tend to have more visible scars, the gravitational forces compounding poor lymphatic drainage towards the axilla may make heterotopic transfer more favorable.

In the immediate postoperative period, patients will remain admitted for monitoring with their extremity elevated. Patients are advised to keep their extremity elevated as tolerated for the first couple of weeks. For patients undergoing orthotopic VLNT, short-stretch wraps can be applied immediately from the fingertips to the proximal arm, as the flap and site of microsurgical anastomosis will not be affected. For patients undergoing heterotopic transplant into the forearm or wrist, any compression, including wraps, is avoided to allow the flap to heal and incorporate. Compression with short-stretch wraps and CDT are resumed after 3–4 weeks.

**Figure 9:**
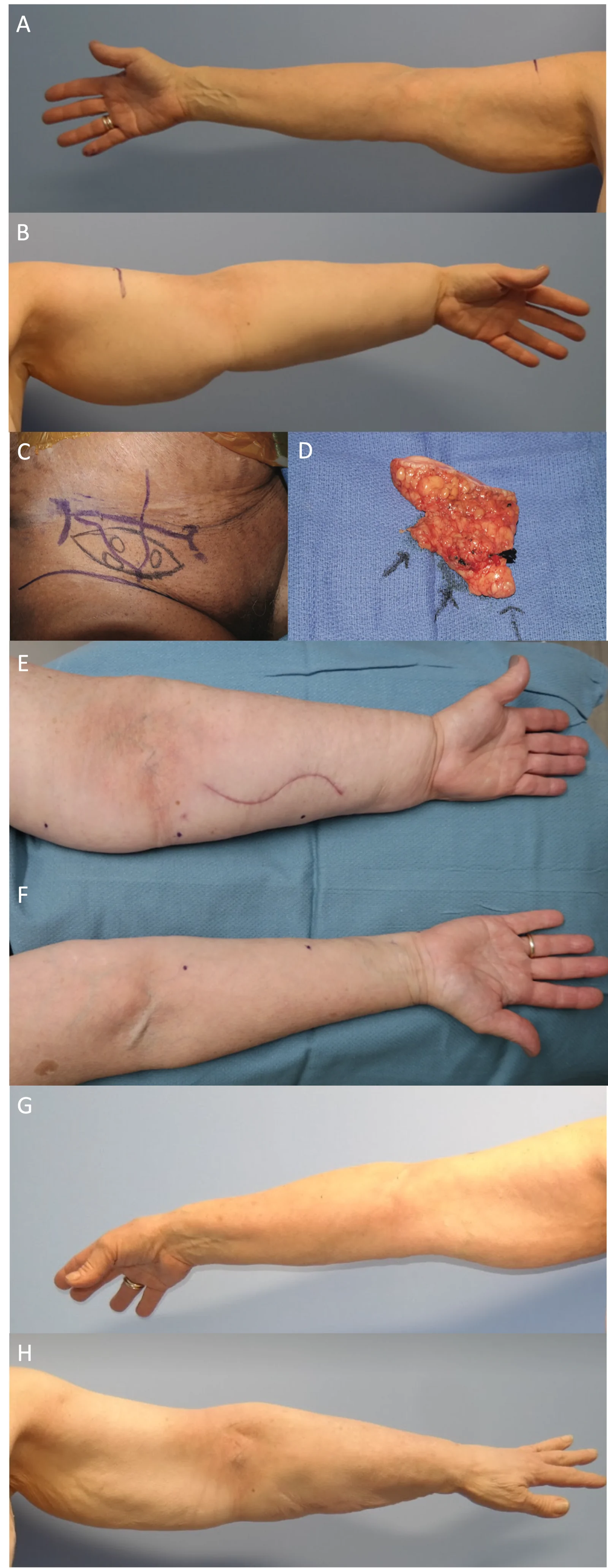
Patient 7 preoperative photographs of a patient with a 10-year history of left upper extremity breast cancer-related lymphedema after axillary lymph node dissection. Volume differential is 2050 mL between the (**A**) right and (**B**) left limbs. (**C**) Vascularized lymph node transfer (VLNT) harvested from the right groin for transfer to the left forearm. Preoperative markings of the groin donor site. (**D**) Harvested lymph node flap with arrows demoting lymph nodes within the flap. (**E**) Postoperative photographs of the left and (**F**) right (bottom) extremities 2 months after vascularized lymph node transfer to the left forearm. (**G**) Postoperative photographs of the right and (**H**) left arms one year after vascularized lymph node transfer to the left upper extremity. Volume differential is 1250 mL between the (**G**) right and (**H**) left limbs. Clinical images are original photographs from the senior authors’ practices.

VLNT has demonstrated substantial benefits in reducing limb volumes and improving symptoms in patients with BCRL (Figure 9). Chang et al. reported a mean volume reduction of 46% in patients who underwent latissimus dorsi VLNT for lymphedema, for both upper and lower extremity lymphedema cases [81]. Ranniko reported a statistically significant mean arm circumference reduction of 2.5 cm at 3 years postoperatively, along with fewer episodes of cellulitis and improved pain and limb function [82]. Combined simultaneous VLNT and breast reconstructions have also shown promise. Nguyen et al. reported an improvement in mean differential limb volume from 21% preoperatively to 10% at 12 months, with no flap loss and only minor complications [83]. A systematic review further demonstrated that among patients who underwent concomitant scar release with VLNT, 90% experienced subjective improvement in lymphedema symptoms compared to those who did not. Additionally, compression garment discontinuation was greater in those who underwent scar release [79].

Quality-of-life outcomes following VLNT were also favorable. Patel et al. reported significant improvement in functional outcomes as early as 1 month postoperatively. Positive functional outcomes were sustained through 1 year, with score increases ranging from 7.8 to 18.6 points across all domains [84]. Cheng et al. found that patients who underwent both groin and submental VLNT for upper extremity lymphedema achieved a 24.4% circumferential reduction, experienced 2.8 fewer episodes of cellulitis, and reported significant quality-of-life improvements at 1 year [84]. In another cohort of 25 patients with stage I-II BCRL treated with groin-based VLNT, 84% demonstrated significant improvements in Upper Limb Lymphedema-27 scores, with a mean improvement in scores by 18 points [85]. Similarly, Gratzon et al. reported statistically significant improvements across all LYMQoL domains in 50 patients who underwent orthotopic VLNT, with each domain improving by at least 5 points at 1 year. Episodes of cellulitis also decreased [86].

3. Adjunct Therapies and Emerging Technologies

Adjunct therapies for the treatment of lymphedema are promising with several ongoing preclinical studies being conducted to test the safety and efficacy in humans. These therapies aim to promote lymphangiogenesis and include implantable devices [87,88], gene-based therapies [89], cell-based therapies [90] and drug-based therapies [91]. In our practice, we use an aligned nanofibrillar collagen scaffold as an adjunct in patients with late-stage lymphedema who are to undergo physiologic procedures. We have also used robotic microsurgery/supermicrosurgery to perform LVB and VLNT.

4. Aligned Nanofibrillar Collagen Scaffold

Aligned nanofibrillar collagen scaffold (ANCS) is a biodegradable, biocompatible, multi-luminal surgical mesh ribbon that is used as an adjunct surgical implant for the treatment of secondary lymphedema [88]. Intraoperatively, ANCS can be tunneled subcutaneously to provide a structural guide for lymphangiogenesis as an adjunct to physiologic lymphatic reconstruction. Early research on ANCS has demonstrated improvement in limb volume and lymphatic drainage following ANCS placement [88,92]. Despite these positive clinical findings, quality-of-life outcomes following ANCS implantation have not been well characterized in the literature.

5. Robot-Assisted Microsurgery and Supermicrosurgery

Robot-assisted microsurgery is an emerging field in lymphatic reconstruction. Robotic platforms such as MicroSure’s MUSA (MicroSure, Eindhoven, The Netherlands) and the Symani Surgical System (MMI, Pisa, Italy) were designed to overcome challenges in microsurgery and supermicrosurgery, including physiologic tremor and ergonomic fatigue [93]. We have used the Symani Surgical System in our practice when performing LVBs and VLNTs. Early studies of robot-assisted LVBs have reported reductions in limb volume compared to baseline, with decreases of 7.6% in the upper extremity and 1.4% in the lower extremity [94,95]. Complication rates were also minimal [94]. Robot-assisted VLNTs have also demonstrated low complication and high flap survival rates [95,96]. Although outcomes were comparable to manual LVBs, surgeons report slightly lower satisfaction scores when performing robotic LVBs [94]. Authors cite limited haptic feedback, instrument delay and longer operative times as impacting satisfaction [94]. A limitation of the Symani Surgical System is the associated learning curve. In our experience, adoption of the robotic platform initially increases operative time, which then becomes comparable to manual performance as surgeons become familiar with robotic instrumentation and workflow.

##### Excisional Techniques

Excisional techniques such as suction-assisted lipectomy and direct excision are indicated in patients with later-stage lymphedema with primarily fibrofatty (solid) tissue deposition. These procedures, especially direct excision, are generally reserved for patients with ISL stage II–III lymphedema, who are not candidates for other more conservative treatments.

1. Lymphatic-Sparing Suction-Assisted Protein Lipectomy (SAPL)

In patients with predominantly fibrofatty or combination fluid and solid volume excess, SAPL can provide substantial limb volume reduction and symptom relief [97,98]. SAPL involves liposuction of the affected extremity to break up fibrous tissue and remove excess volume. Areas of fibrofatty deposition are targeted for liposuction, with the posterior arm being a common area with excess volume deposition. To minimize blood loss, tumescence with epinephrine is infiltrated into the areas to be liposuctioned, and a tourniquet is utilized. To avoid damaging functional lymphatic channels during the procedure, intraoperative lymphatic mapping with intradermal ICG is injected into the web spaces, and areas with functioning lymphatics are marked out and avoided. It is also believed that using longitudinal passes with the liposuction cannulas will further reduce the chances of inadvertent lymphatic disruption.

SAPL may be performed in conjunction with physiologic procedures such as VLNT and LVB to address both fluid and fibrofatty components of lymphedema. The sequencing of these approaches, as described by Brazio/Nguyen [99] and Salibian/Yu/Patel [26], is tailored to the clinical presentation.

In patients with advanced, fibrofatty disease and significant volume excess, liposuction is performed to debulk the limb, followed by a staged physiologic procedure to remove fluid excess if intact lymphatic channels are still present [99]. Debulking has been shown to improve lymphatic flow on lymphoscintigraphy, as demonstrated by decreased dermal backflow [26]. Secondary physiologic procedures further reduce episodes of cellulitis and pitting edema, and help wean compression—outcomes not achievable through debulking alone [26]. Identification of functional lymphatic channels can be more challenging in areas of prior liposuction, which is why proximal LVB is favored in staged reductive and physiologic procedures [26].

In patients with pitting, fluid-predominant edema, a physiologic procedure is performed first, followed by staged SAPL if adequate volume reduction is not achieved [99]. Given the progressive nature of lymphatic disease, SAPL may be delayed for 6 months after a physiologic procedure to assess a response [26].

In patients with minimal response to an initial physiologic procedure and persistent fluid burden, a secondary physiologic procedure may be considered [26]. Performing a VLNT after an LVB is common; however, LVB after a VLNT may be more challenging due to the lack of functional lymphatic channels [26]. In patients with a good response to LVB, additional bypasses have demonstrated a synergistic effect and have been associated with improved outcomes [26].

For patients with a mixed edema presentation, simultaneous physiologic and liposuction procedures may be performed, although care must be taken to avoid disrupting the bypass or nodal transfer sites [26,99]. In these cases, physiologic procedures are performed first distally, and suctioning is reserved for the last portion of the surgery so the areas of LVB or VLNT can be marked out and avoided [99].

Postoperative compression therapy remains essential after debulking procedures. Custom compression garments are ideally ordered preoperatively and used immediately after surgery [100]. Alternatively, short-stretch wraps may be used until the initiation of lymphedema therapy. Early transition to CDT is critical as strict adherence to compression is paramount to counteract the rebound swelling that occurs after liposuction and maintain reduced limb volumes over the long term. Therefore, compression therapy remains an important long-term adjunct to treatment rather than a temporary measure, especially following debulking procedures.

**Figure 10:**
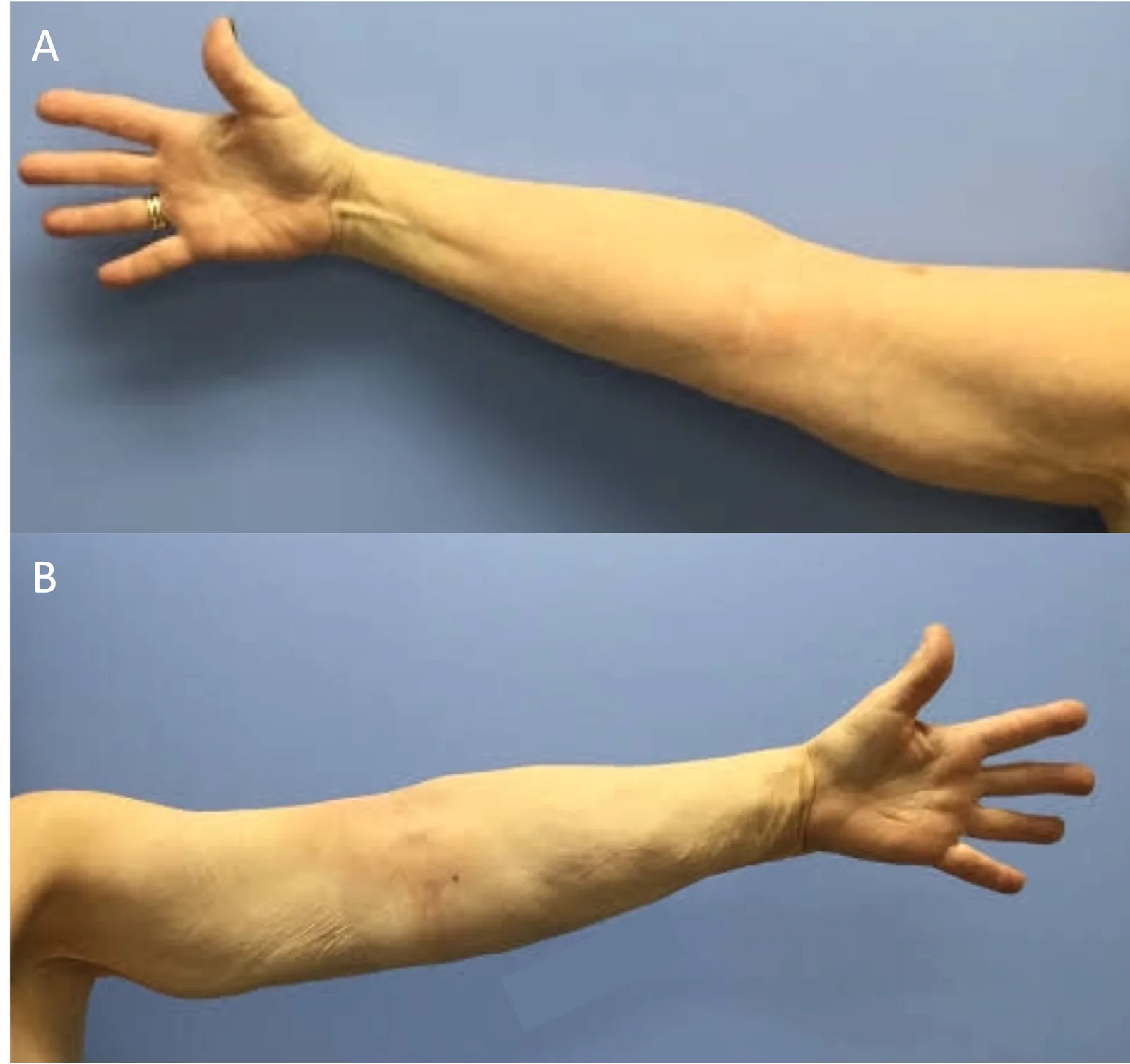
Postoperative photographs of the (**A**) right and (**B**) left arms one year after undergoing liposuction and compliance with compression, with volume differential of 115 mL between the two limbs. Clinical images are original photographs from the senior authors’ practices.

Limb volume outcomes respond to the type or combination of treatment measures applied. Compression therapy alone is often insufficient for control in advanced disease. In contrast, liposuction combined with controlled compression therapy (CCT) has demonstrated significant reductions in excess limb volume (Figure 10). In patients undergoing lipectomy for secondary lymphedema, Greene et al. reported a mean reduction in limb volume of 73% at 3.1 years follow-up [101]. Patients in this cohort also reported subjective improvements in limb function, improved quality of life, and fewer episodes of cellulitis [101]. Liposuction and CCT has been shown to reduce percent excess volume by 26.59% and decrease the incidence of severe cellulitis postoperatively to 10% [102]. When comparing the effects of this combined approach to compression therapy alone, Brorson and Svensson demonstrated that at a 1 year follow-up, patients treated with liposuction followed by CCT achieved a relative volume reduction of 113%, compared to 47% in those managed with compression therapy alone [103]. When combined with physiologic procedures, liposuction helps address both the fibrofatty tissue burden and impaired lymphatic drainage [99]. Patients who underwent simultaneous procedures, performing both a physiologic procedure and liposuction in a single anesthetic event, demonstrated the smallest excess volume reduction of 82%. In contrast, patients treated with a staged approach, specifically when liposuction was performed first followed by a physiologic procedure, demonstrated up to 106% reduction in excess volume after the second stage [99]. In the two-stage procedures, the liposuction-first approach may be more effective for patients with significant volume excess requiring debulking [99]. The physiologic-first approach is better suited for fluid-predominant disease [99]. The simultaneous approach offers a balanced option for mixed presentations and avoids multiple surgeries for these patients [99].

In addition to objective volume reduction, liposuction is associated with improvements in patient-reported outcomes. Bustos et al. reported that more than 90% of patients undergoing power-assisted liposuction for upper extremity lymphedema experienced improvement or resolution of lymphedema symptoms [104]. Furthermore, 85% reported improvement in tightness, and 68% noted improvement or resolution of numbness and tingling [104]. Hoffner et al. assessed HRQoL using the 36-Item Short Form Health Survey (SF-36) in 60 upper extremity lymphedema patients who underwent liposuction and compression therapy [105]. Both mental and physical component scores on the SF-36 increased by at least 3 points at a 1 year follow-up [105].

2. Direct Excision

The goal of this debulking procedure is to remove excess skin and subcutaneous tissue to reduce overall limb volume. These procedures tend to have higher morbidity and are thus reserved for patients with more severe, advanced-stage lymphedema and fibrosis. The general goal of direct excision is for symptomatic relief via debulking of the extremity, though there is some research to support the idea of increasing the proximity between the superficial and deep lymphatics, and potentially improving lymphatic flow by bridging the superficial and deep systems [28].

The Charles procedure was one of the first excisional options for patients with late-stage lymphedema. The original technique involved excision of the skin and redundant subcutaneous tissue to the deep fascia, followed by immediate coverage with a skin graft [106]. Due to the high morbidity of the surgery, with complications including significant blood loss, graft loss, infections, and exacerbation of lymphedema, the Charles procedure is rarely performed and is reserved as a last option for patients with debilitating end-stage disease who are not candidates for other debulking procedures [107]. Subsequent direct excisional techniques, such as the Homans procedure, involve raising longitudinal flaps of skin to remove the underlying subcutaneous tissue and deep fascia [108,109]. Mousavi reported that in patients who underwent removal of the subcutaneous tissue and deep fascia for lower limb lymphedema, 87.8% were symptom-free and satisfied with their outcomes at 1 year [108]. Complications among this cohort were relatively low, including seroma formation in 5.4% of patients, wound infection in 3% and flap ischemia in 2.3% [108]. Other techniques incorporate elliptical skin excision patterns to remove skin in addition to fibrofatty tissue. Tailor tacking before resecting skin helps to confirm the amount of skin to be safely resected while avoiding excess tension along the closure line. Another modification of the Charles procedure, called radical reduction with preservation of perforators (RRPP), involves using knowledge of angiosomes and skin perforator territories to excise large amounts of skin and soft tissue while preserving adequate vascularity in the remaining skin. Using RRPP, Chen reported an average overall reduction in lymphedema of 52% in patients with lower extremity lymphedema. His group also published a study combining VLNT with RRPP direct excision, showing a 75% limb volume reduction for upper extremity lymphedema and a significant (2.7-fold) improvement in patient quality of life based on the LYMQoL [110].

## 4. Conclusions

Breast cancer-related lymphedema (BCRL) remains a feared complication after breast cancer treatment that has substantial physical and socioeconomic consequences. As patients outlive their cancer diagnosis, clinicians are tasked with optimizing their long-term quality of life and reducing morbidity associated with BCRL. Although there is no cure for BCRL, advances in diagnostic tools, including lymphoscintigraphy, ICG lymphography, MRL, and UHFUS, help to characterize and diagnose the disease. The diagnostic algorithm presented in this review brings these tools together and provides a practical approach to the workup of patients with suspected BCRL. After diagnosis, CDT and compression remain the foundation of treatment. These therapies also remain important throughout surgical management.

Surgical treatment requires an individualized approach. Patients should be medically optimized and undergo appropriate conservative treatment prior to pursuing surgery. Surgeons can use disease stage and tissue composition to determine whether a physiologic or reductive approach is most appropriate. Functional lymphatic anatomy further guides the choice of physiologic procedure. LVB can improve lymphatic drainage in patients who retain functional lymphatic channels. VLNT provides another physiologic option when few functional channels remain. Recurrent cellulitis may also favor VLNT. SAPL can reduce excess volume in patients with predominantly fibrofatty disease. Direct excision remains an option for patients with severe disease who are not candidates for less invasive approaches. Patients with mixed disease may benefit from staged or combined physiologic and reductive procedures. The surgical algorithm presented in this review incorporates these principles and provides a framework for treatment selection. This approach recognizes that BCRL changes over time. Treatment must therefore reflect the characteristics of the disease at the time of intervention.

Advances in lymphatic surgery continue to expand the available treatment options. Adjunct therapies such as ANCS may promote lymphangiogenesis after physiologic reconstruction. Emerging technologies may also improve how surgeons perform lymphatic procedures. Robotic-assisted microsurgery may facilitate supermicrosurgical anastomoses through motion scaling and tremor reduction. However, these newer approaches require further study before their role in routine BCRL management becomes clear. Oncologic treatment also remains an important consideration. Clinicians generally consider delayed lymphatic reconstruction after patients complete the necessary oncologic treatment. The effects of systemic breast cancer therapies on the development and progression of BCRL remain incompletely understood. Future studies should better define these relationships.

Despite advances in diagnosis and treatment, the current literature has important limitations. Studies differ in patient selection and disease staging. Investigators also use different outcome measures and follow-up periods. These differences limit comparisons between treatment approaches and make standardized surgical indications difficult to establish. Future research should prioritize prospective head-to-head trials that compare LVB with VLNT and reductive approaches. Studies should also evaluate staged and combined treatment strategies. Longer-term follow-up will help determine the durability of these procedures. Standardized reporting of limb volume and patient-reported outcomes will improve comparisons across studies.

BCRL management ultimately requires a multidisciplinary and patient-specific approach. No single treatment addresses every stage or presentation of the disease. The diagnostic and surgical algorithms presented in this review connect disease evaluation with treatment selection and provide a framework for clinical decision-making. As lymphatic imaging and surgical techniques continue to evolve, stronger comparative evidence will help refine these algorithms. These advances may allow clinicians to select treatments more precisely and improve long-term outcomes for patients with BCRL.

## Data Availability

No new data were created or analyzed in this study. Data sharing is not applicable to this article.

## Abbreviations

The following abbreviations are used in this manuscript:

BCRL	Breast Cancer-Related Lymphedema
LVB	Lymphovenous Bypass
VLNT	Vascularized Lymph Node Transfer
ALND	Axillary Lymph Node Dissection
SLNB	Sentinel Lymph Node Biopsy
HRQoL	Health-Related Quality of Life
BMI	Body Mass Index
ISL	International Society of Lymphology
DVT	Deep Vein Thrombosis
ICG	Indocyanine Green
MRL	Magnetic Resonance Imaging Lymphography
UHFUS	Ultra-High-Frequency Ultrasound
CDT	Complete Decongestive Therapy
MLD	Manual Lymphatic Drainage
ILR	Immediate lymphatic reconstruction
ANCS	Aligned Nanofibrillar Collagen Scaffold
ECM	Extracellular Matrix
SAPL	Suction-Assisted Protein Lipectomy
CCT	Controlled Compression Therapy
RRPP	Radical Reduction with Preservation of Perforators
LYMQoL	Lymphedema Quality of Life
